# miR-31 Links Lipid Metabolism and Cell Apoptosis in Bacteria-Challenged *Apostichopus japonicus via* Targeting CTRP9

**DOI:** 10.3389/fimmu.2017.00263

**Published:** 2017-03-13

**Authors:** Yina Shao, Chenghua Li, Wei Xu, Pengjuan Zhang, Weiwei Zhang, Xuelin Zhao

**Affiliations:** ^1^School of Marine Sciences, Ningbo University, Ningbo, China; ^2^Agricultural Center, Louisiana State University, Baton Rouge, LA, USA

**Keywords:** *Apostichopus japonicus*, miR-31, complement C1q tumor necrosis factor-related protein 9, lipidomics, ceramide, apoptosis

## Abstract

The biological functions of microRNAs (miRNAs) have been studied in a number of eukaryotic species. Recent studies on vertebrate animals have demonstrated critical roles of miRNA in immune and metabolic activities. However, studies on the functions of miRNA in invertebrates are very limited. Here, we demonstrated that miR-31 from *Apostichopus japonicus* disrupts the balance of lipid metabolism, thus resulting in cell apoptosis by targeting complement C1q tumor necrosis factor-related protein 9 (AjCTRP9), a novel adipokine with pleiotropic functions in immunity and metabolism. Lipidomic analysis suggested that the intercellular lipid metabolites were markedly altered, and three ceramide (Cer) species synchronously increased in the AjCTRP9-silenced coelomocytes. Moreover, exogenous Cer exposure significantly induced apoptosis in the coelomocytes *in vivo*, in agreement with findings from miR-31 mimic- or *AjCTRP9* small-interfering RNA-transfected coelomocytes. Furthermore, we found that the imbalance in sphingolipid metabolism triggered by the overproduction of Cers ultimately resulted in the activation of the apoptosis initiator caspase-8 and executioner caspase-3. Our findings provide the first direct evidence that miR-31 negatively modulates the expression of *AjCTRP9* and disturbance of Cer channels, thus leading to caspase-3- and caspase-8-dependent apoptosis, during the interactions between pathogens and host.

## Introduction

After the invasion of pathogens, the innate immune response reacts immediately as the first barrier to defend the host. Various cellular and humoral defense pathways are simultaneously activated through different signaling networks. Efforts have been made to identify the roles of immune-related molecules during immune responses. However, studies on the regulatory networks of these molecules and their interactions in host defense processes are lacking. Emerging studies deciphering the various cellular and signaling networks linking the immune defense with metabolism have contributed to the understanding of disease pathogenesis and the development of therapeutic strategies (1). It is therefore no surprise that the dissection of the interface connecting the immune system to metabolic pathways including glycolysis, lipid, and amino acid metabolism has recently gained interest (2, 3). This metabolic regulation is largely controlled by extracellular signals that direct the uptake, storage, and utilization of substrates (glucose, amino acids, and fatty acids) under immune responses. Among them, lipid metabolism has been shown to be closely related to the immune response, because the lipids of the membrane bilayer form the first line of barrier between a cell and its environment (4), and lipid metabolism generates a variety of bioactive signaling molecules, including phosphatidic acid, triacylglycerol (TAG), sphingosine, and ceramide (Cer), which participate in the regulation of many cellular processes such as cell growth, membrane homeostasis, inflammation, and apoptosis (5). Imbalances in cellular lipid metabolism increase the susceptibility of the host to pathogenic invasions and ultimately lead to cell death in the host (6, 7). Therefore, the regulation of lipid metabolism may also change the host immune response to pathogenic challenge. Manipulation of lipid metabolism pathways may contribute to the enhancement of host immune systems against pathogens.

microRNAs (miRNAs) are highly conserved small non-coding RNAs (~22 nucleotides) that regulate gene expression primarily by binding to the 3’-untranslated regions of target mRNAs, thus decreasing target protein synthesis and sometimes resulting in mRNA degradation (8, 9). As one of the most abundant classes of gene regulators, miRNAs are involved in many biological processes, such as cell proliferation (10), tumorigenesis (11), apoptosis (12), and inflammation (13). Recent studies have indicated that miRNAs also play novel roles in regulating metabolic pathways through the control of target genes involved in metabolic processes (14, 15), and especially in controlling lipid metabolic homeostasis and host inflammatory responses in cardiovascular diseases (16, 17).

To date, more than 30 miRNAs have been identified to play crucial roles in the regulation of lipid metabolism through various pathways, such as fatty acid oxidation, and biosynthesis of fatty acids, cholesterol, and TAG (5, 18, 19). The first evidence linking specific miRNAs to critical pathways in lipid metabolism has been found in the fruit fly *Drosophila melanogaster*, thus indicating that dme-miR-14 regulates lipid metabolism by downregulation of the production of TAG and diacylglycerol (20). Later, another miRNA, miR-33, which has two isoforms (miR-33a and miR-33b) and is highly conserved across species, was identified from a number of cell types including macrophages, hepatocytes, and endothelial cells (21). The overexpression of miR-33a/b decreases both fatty acid β-oxidation and insulin signaling in radioactively labeled hepatic cell lines, whereas the inhibition of endogenous miR-33a/b increases these two metabolic pathways, thereby suggesting that miR-33 plays important roles in regulating lipid homeostasis in concert with the host genes (22). Moreover, a study by Ouimet et al. has revealed that macrophage-specific miR-33 deletion increases oxidative respiration and decreases atherosclerosis progression by promoting the M2 macrophage polarization-associated gene profile, thus suggesting that silencing miR-33 may be an effective therapeutic approach in the treatment of chronic inflammatory diseases (17).

Multiple miRNAs may also share the same target mRNA in the regulating lipid metabolism. Both miR-370 and miR-122 directly target the transcription factor sterol regulatory element binding protein 1c in Hep2G cells, thereby regulating fatty acid and triglyceride biosynthesis (23). Other miRNAs, such as miR-130 and miR-335, are also important players in the regulation of lipid metabolism (24, 25). Although connections between lipid metabolism and the host inflammatory responses through miRNA have been studied (26), the molecular mechanisms of how the miRNAs modulate lipid metabolism in response to pathogenic diseases remain unclear. Systemic studies on the network of lipid metabolism and host defenses in cells are still needed.

The invertebrate sea cucumber, *Apostichopus japonicus* (Echinodermata, Holothuroidea), has an innate immune system and is one of the most important marine economic species in the Chinese aquaculture. Unfortunately, the natural sources of *A. japonicus* in China have declined drastically, owing to outbreaks of various viral and bacterial diseases (27). Among these diseases, skin ulceration syndrome (SUS) causes high infectious and lethal rates and has received substantial attention (27, 28). In our previous work, we have found that miR-31 and miR-2008 expression levels are markedly increased in sea cucumbers with SUS by high-throughput analyses, thus indicating that these molecules play crucial roles in this host–pathogen interaction (29). Our other work has also demonstrated that the samples with SUS exhibit significant metabolic differences compared with the metabolic profiles from healthy *A. japonicus* (30). Undoubtedly, miRNAs might play essential roles in mediating the innate immunity and metabolic pathways in the sea cucumbers with SUS. The connection between miRNAs and the innate immune response is largely unknown to the best of our knowledge, especially in non-model organisms such as the sea cucumber. The present study aimed at exploring the potential of regulation of the complement c1q tumor necrosis factor-related protein 9 gene (*AjCTRP9*), a novel adipocyte-derived cytokine, by miR-31. CTRP9 is an adipokine that has pleiotropic functions in immunity and metabolism (31, 32) and was selected as a promising target for functional validation. Our results clearly demonstrated that miR-31, a multifunctional regulatory molecule, negatively modulates the expression of *AjCTRP9* and induces cell apoptosis by disturbing the lipid metabolism balance, thus resulting in the overproduction of Cers. The findings of the current study provide new insights into the mechanisms of pathogenesis of infectious diseases in *A. japonicus* from the perspective of miRNA-mediated cross-talk between the immune response and lipid metabolism.

## Materials and Methods

### Prediction of miR-31 Targets

The potential targets of miR-31 were predicted by using the miRanda v3.01 toolbox by screening of our previous transcriptome data (33, 34). The parameters were set as follows: single–residue pair scores less than the threshold value of 90 and a minimum free energy lower than −17 kcal/mol. Subsequently, the promising candidate metabolism-associated genes with the highest scores and lowest free energy values was selected as potential targets for further analysis.

### Animals and Challenge Experiments

Healthy adult sea cucumbers *A. japonicus* (weighing 150 ± 15 g) were obtained from the Dalian Pacific Aquaculture Company (Dalian, China) and acclimatized in 30 L aerated natural seawater (salinity 28, temperature 16°C) for 3 days. The pathogen *Vibrio splendidus* was initially isolated from sea cucumbers with SUS from the indoor farms of Jinzhou Hatchery in May 2013, preserved in glycerol, and stored at −80°C. For the immune challenge, the sea cucumbers were randomly divided into five tanks, each containing 10 individuals. Four experimental groups were immersed with live *V. splendidus* at a final concentration of 10^7^ CFU mL^−1^. An identical number of individuals in another tank were kept as controls. The coelomic fluids from five individuals were collected after exposure for 0, 6, 24, 48, and 72 h and then centrifuged at 800 *g* for 5 min to harvest the coelomocytes for the time course expression analysis.

### Cloning and Characterization of Full-Length CTRP9 from *A. japonicus*

The partial sequence of the *CTRP9* gene was generated by screening the *A. japonicus* transcriptome database (34). BLASTx analysis of the fragment revealed that the sequence contained the complete 5’-end compared with that of other reported sequences. No polyA tail was detected in the *AjCTRP*9 fragment. Therefore, gene-specific primers for *AjCTRP9* (Table S1 in Supplementary Material) were designed on the basis of the acquired unigenes, and the full-length cDNA sequence was subsequently cloned using a 3’-Full RACE Kit (TaKaRa), by following the manufacturer’s instructions. The desired PCR products were purified and then cloned into the pMD19-T vector. Three positive clones for each product were sequenced at Sangon (Shanghai, China). Subsequently, the full-length cDNA sequence of *AjCTRP9* was analyzed using the BLAST program at National Center for Biotechnology Information^1^ and the deduced amino acid sequence was analyzed with the expert protein analysis system.^2^ The signal peptide protein domain features were predicted with the Simple Modular Architecture Research Tool.^3^ The molecular mass and theoretical isoelectric point (*pI*) of the protein were calculated on the basis of the deduced amino acid sequence with the ProtParam tool.^4^ Multiple alignment analysis of each protein was performed using the ClustalW2 multiple alignment program.^5^

### 3’-UTR Luciferase Reporter Assays

The complete 3’-UTR of wild-type *AjCTRP*9 containing the putative target sites of miR-31 was amplified with gene-specific primers (Table S1 in Supplementary Material) to generate fragments and cloned into the pMD19-T simple vector (TaKaRa). The putative miRNA binding sites were mutated with a PCR approach with mutagenic primers (Table S1 in Supplementary Material) and served as the mutant *AjCTRP*9. The resulting clones were then digested with *Mlu*I and *Hin*dIII and inserted into *Mlu*I/*Hin*dIII-digested pMIR-REPORT luciferase plasmid (Promega, USA). These plasmids were sequenced to ensure correct orientation, and each plasmid was purified with an EZNA™ Plasmid Mini Kit (OMEGA, USA). For the transfection experiment, HEK293-T cells were seeded in 96-well plates with 100 μL medium and grown to approximately 70% confluence. Then, two solutions were added to each well as follows: the first solution contained 0.2 μg of pMIR-REPORT with either the wild-type or mutated *AjCTRP9* 3’-UTR and 0.01 μg of pRL-CMV constructs (the internal control) with 0.25 μL of the Lipofectamine 2000 transfection reagent (Invitrogen, USA). The second solution contained 100 nM miR-31 mimics (Table S1 in Supplementary Material) and 0.25 μL of the transfection reagent. Twenty-five microliters of each solution, diluted in the medium, were mixed before being added to the wells, and the plates were incubated at room temperature for 20 min. Subsequently, the solutions were replaced with 50 μL of medium in each well. The cells co-transfected with 100 nM miR-31 mimics served as the negative control (NC) (NCM; Table S1 in Supplementary Material), and wild-type or mutated constructs served as the positive control. After 48 h of transfection, the cells were prepared for luciferase activity assays using a dual-luciferase reporter assay kit (Promega, USA). The luciferase signal was expressed as a ratio of firefly to Renilla luciferase activities. All of the experiments were performed in six replicates.

### Primary Coelomocytes Culture and LPS Exposure

Sea cucumbers (weighing 150 ± 15 g) were dissected with sterilized scissors on ice, as previously described (35, 36). In brief, the coelomic fluids were filtered through a 300-mesh CellCribble to remove large tissue debris, mixed with the anticoagulant solution (0.02 M EGTA, 0.48 M NaCl, 0.019 M KCl, 0.068 M Tri–HCl, pH 7.6) in a 1:1 (V:V) ratio, and then centrifuged at 800 *g*, 16°C for 10 min. The harvested cells were washed twice with isotonic buffer (0.001 M EGTA, 0.53 M NaCl, 0.01 M Tris–HCl, pH 7.6) and resuspended in Leiboviz’s L-15 cell culture medium (Invitrogen, USA) containing penicillin (100 U mL^−1^) and streptomycin sulfate (100 mg mL^−1^); NaCl (0.39 M) was added to adjust the osmotic pressure. The cell suspension was diluted to 10^6^ cells mL^−1^ and transferred into 24-well culture microplates and incubated at 16°C for 12 h before exposure to lipopolysaccharides (Sigma, USA). For the LPS challenge, the cells were stimulated with 10 μg mL^−1^ LPS for 0, 3, 6, 12, and 24 h. The untreated cells served as the control. The cells were collected in TRIzol and used for expression analysis.

### Expression Analysis of miR-31 and mRNAs under Immune Challenge

The expression levels of mRNAs and miR-31 were analyzed using a Rotor-Gene 6000 real-time PCR detection system. Total RNA was isolated from the coelomocytes using TRIzol (Takara), and cDNA was synthesized using a PrimeScript miRNA RT-PCR Kit (Takara). The primers used are listed in Table S1 in Supplementary Material. RNU6B and *Aj*β*-actin* served as internal controls to normalize the miRNA or the target, respectively, for quantification. Each amplification was carried out in a 20 μL reaction volume containing 8 μL of the 1:50 diluted cDNA, 1 μL of each of the primers, and 10 μL of SYBR Green Mix (Takara). The reaction mixture was incubated for 5 min at 95°C, and this was followed by 40 amplification cycles of 15 s at 95°C, 20 s at 60°C, and 20 s at 72°C. The 2^−ΔΔCT^ method was used to analyze the relative expression levels of both miRNA and mRNA (37), and the values obtained denoted the fold difference relative to the calibrator. The data are presented as the relative expression levels (mean ± SD, *n* = 5). Differences were considered significant at *p* < 0.05.

### Functional Analysis of miR-31 *In Vivo*

The miR-31 mimics and inhibitor with the 2’-OMe modification were synthesized by GenePharma (Shanghai, China) and are shown in Table S1 in Supplementary Material. These reagents were dissolved in RNase-free water to obtain a 20 μM working solution. We mixed 10 μL (2 μM) of miR-31 mimics or inhibitor from each working solution with 10 μL of transfection reagent and 80 μL of phosphate buffered solution (PBS) at pH 7.6 to serve as the transfection solution. Ten sea cucumbers (100 ± 10 g) were infected with 100 μL of the above transfection solution. Twenty-four hours after the injection, the control and treated coelomocytes were collected for further RNA and protein extraction.

### Silencing of AjCTRP9 *In Vivo*

Three specific small-interfering RNAs (siRNAs) targeting *AjCTRP9* with the extra 2’-OMe modification were designed and synthesized by GenePharma (Shanghai, China). Another siRNA (NC) that did not target any of the genes from the sea cucumber transcriptome data served as a NC. The detailed sequences are shown in Table S1 in Supplementary Material. Each siRNA and the NC siRNA were dissolved in RNase-free water to obtain a 20 μM working solution. For RNA interference, we mixed 10 μL of each siRNA or the NC siRNA with 10 μL of transfection reagent and 80 μL of PBS to serve as the transfection solution. Ten sea cucumbers (100 ± 10 g) were injected with 100 μL of the transfection solution into the tentacles. Twenty-four hours after the injection, the control and treated coelomocytes were collected for further RNA, protein, and lipid extraction. *AjCTRP9* expression was analyzed as described below, and the interference efficiency was taken as the average after three siRNA transfections.

### AjCTRP9 Recombinant Protein Expression and Polyclonal Antibody Preparation

The partial cDNA sequence of *AjCTRP9* was cloned with specific primers (Table S1 in Supplementary Material) then double digested with *Eco*RI and *Not*I and ligated into the vector pGEX-4T-2 (GE, USA). Ajβ-actin was used as a control, as described in our previous work (38). The recombinant plasmid (pGEX-4T-2-AjCTRP9) was transformed into *Escherichia coli* cells of Transetta (DE3) (TransGen, China), and the DNA was sequenced to ensure correct orientation. The positive transformants were subsequently incubated in LB medium containing 50 μg mL^−1^ of ampicillin at 37°C and 200 rpm. Then, the expression of the GST-tagged fusion protein was induced in pGEX-4T-2-AjCTRP9 with 1 mM isopropyl-b-d-thiogalactopyranoside (IPTG) at 37°C for 4 h, and the protein was collected after cells were disrupted with ultrasonic waves. The GST-Sefinose™ Resin was used for the purification, according to the manufacturer’s protocol (Sangon, China). The concentration of the purified soluble protein was quantified with a Protein Assay Kit (Sangon, China). The soluble target protein, after dialysis, was injected into a 4-week-old mouse to acquire polyclonal antibody according to a previously described protocol (38).

### Western Blot Analysis

For the western blot assay, the samples were washed twice in ice-cold PBS and lysed with lysis buffer (Beyotime Biotechnology, China). After lysis, the cells were centrifuged at 10,000 *g* for 5 min, the supernatant containing the total protein was collected, and the protein concentration was measured using a BCA Protein Assay Kit (Sangon, China). Each sample (25 μg) was resolved with a 12% SDS-PAGE gel and then transferred to polyvinylidene fluoride membranes at 300 mA for 1 h (Millipore, USA) in transfer buffer (25 mM Tris, 192 mM Glycine, 20% [v/v] E-OH). After being blocked with 5% fat-free dry milk in TBST (25 mM Tris–HCl, pH 7.4, 137 mM NaCl, 2.7 mM KCl, 0.1% [v/v] Tween 20) for 1 h at room temperature, the membranes were incubated overnight with AjCTRP9 or Ajβ-actin polyclonal antibodies diluted to 1:500 in 5% fat-free dry milk in TBST. The membranes were washed three times with TBST and subsequently incubated at room temperature for 1 h with goat anti-mouse (Beyotime Biotechnology, China) diluted at 1:3,000 in 5% fat-free dry milk in TBST. After being washed three times with TBST for 10 min each, the membranes were incubated in Western Lightning-ECL substrate (Perkin Elmer) before exposure onto X-OMAT AR X-Ray films (Eastman Kodak, Rochester, NY, USA).

### Liquid Chromatography and Mass Spectrometry (LC-MS) Assay after AjCTRP9 Silencing

After *AjCTRP9* silencing, 50 mg of coelomocytes (freeze-dried) was extracted in chloroform/methanol/water (1:2:0.8, v/v/v) containing 0.05% butylated hydroxytoluene (BHT), according to a modified version of the Bligh and Dyer method (39). Briefly, the obtained extract was evaporated on a rotary evaporator, and the lipid residue was redissolved in 1 mL of methanol after centrifugation at 12,000 rpm for 5 min at 4°C. The methanol solution of the lipids was filtered with a 0.22 μm ultrafiltration membrane (Millipore, USA) to remove the undissolved residues and subjected to LC-MS analysis using the previously published standard method (40–42).

Chromatographic separations were performed on a Waters ACQUITY UPLC system using an ACQUITY UPLC BEH C8 analytical column (i.d. × l 2.1 × 100 mm, particle size 1.7 μm). Briefly, lithium acetate (0.01%) and formic acid (15 mM) were added to the mobile phase as the electrolyte for the ESI positive mode analysis, and ammonium hydroxide (15 mM) was added for the negative ion mode analysis. To obtain efficient separation of the total lipids, water/acetonitrile (1:2, v/v) was used as mobile phase A, and acetonitrile/isopropyl alcohol/tetrahydrofuran (1:1:1, v/v/v) was used as mobile phase B. The mobile phase B was changed from 0 to 50% in 15 min, then to 80% in 30 min and held for 2 min and finally to the initial 0 in 2 min and equilibrated for 11 min. The temperature of the sample chamber was set at 4°C, and the injection volume was 5 μL for each analysis. Twenty-five percent of the flow out of the column was subjected to mass spectrometry.

Mass spectrometry was performed on a Waters Q-TOF Premier mass spectrometer operating in both positive and negative ESI modes. The nebulization gas was set to 400 L h^−1^ at a temperature of 300°C, the cone gas was set to 0 L h^−1^, and the source temperature was set to 120°C. For the ESI^+^ mode, the capillary voltage was set at 3.0 kV and the sampling cone voltage was set at 80 V. For the ESI^−^ mode, the capillary voltage was set at 2.5 kV, and the sampling cone voltage was set at 60 V. MS^2^ analysis was performed at a collision energy range of 20–40 V. A time-of-flight (TOF) analyzer was used in a V mode and tuned for maximum resolution (>10,000 resolving power at *m*/*z* 1,000). The instrument was previously calibrated with sodium formate, and the lock mass spray for precise mass determination was set with leucine-enkephalin, thus generating an [M + H]^+^ ion at 556.2771 Da and an [M − H]^−^ ion at 554.2615 Da in the ESI^+^ mode and ESI^−^ mode, respectively. The lock spray frequency was set at 10 s.

The raw data files obtained from ultra-performance liquid chromatography-quadrupole time-of-flight mass spectrometry runs were analyzed using a MassLynx 4.1 data processing system (Waters, Milford, MA, USA). The resulting multivariate dataset consisting of the peak number (based on the retention time and *m*/*z*), sample name, and the normalized peak intensity was exported and analyzed with principal component analysis (PCA), the projection to latent structures was analyzed with discriminant analysis (PLS-DA), and the orthogonal projection to latent structures was analyzed with discriminant analysis (OPLS-DA) using SIMCA-P-12.0 software (Umetrics, Sweden). The loading scatter S-plots and contribution lists were used to describe the candidate markers that were significantly different between the two groups. One-way analysis of variance (ANOVA) was conducted on the *A. japonicus* between the NC and siAjCTRP9 groups to determine the statistical significance (*p* < 0.05) of the candidate markers.

### Cell-Permeable C_6_ Cer Treatment

C_6_ Cer (Biomol, USA), short for C_6_ Cer, was dissolved in anhydrous DMSO to obtain 5 mg mL^−1^ C_6_ Cer stock solution. For Cer treatment, 10 sea cucumbers (100 ± 10 g) were injected with 100 μL of Cer at the concentration of 100 μg mL^−1^. The sea cucumbers in the control group were given an injection of 100 μL diluted DMSO. Twenty-four hours after the injection, the treated and control coelomocytes were collected for further expression analysis.

### Determination of Apoptosis by Flow Cytometry Assay (FACS)

*In vivo* cell apoptosis rates were determined by flow cytometry. The sea cucumbers were injected with miR-31 mimics, miR-31-inhibitor, *AjCTRP9* siRNA, and C_6_ Cer, as described above. The apoptosis rates of the coelomocytes were measured with an Annexin V-FITC Apoptosis Detection Kit (Beyotime Biotechnology, China). For Annexin V/PI staining, the collected coelomocytes were resuspended in 200 μL Annexin V-FITC binding buffer and 5 μL Annexin V-FITC and 10 μL PI were successively added to 10^5^ cells. Finally, the apoptosis rates were detected with a FACScan (BD, USA) after 10 min incubation in the dark at room temperature.

### Measurement of Caspase Activity

Caspase-3/8 activity assays were performed using a Caspase Activity Assay Kit (Beyotime Biotechnology, China). Briefly, the samples were lysed with lysis buffer for 15 min on ice. Then, the cells were centrifuged at 16,000 *g* for 20 min at 4°C, and the supernatant was immediately used for caspase-3/8 enzyme assays. Caspase activity was measured through the cleavage of a colorless substrate Ac-DEVD-*p*NA for caspase-3 and Ac-IETD-*p*NA for caspase-8, thus releasing *p*NA (*p*-nitroaniline). Absorbance was measured at OD_405 nm_ using a microplate reader (Thermo Scientific). The protein concentration of the cells was measured using a Bradford Protein Assay Kit (Beyotime Biotechnology, China). Caspases-3/8 activities were calculated using a standard curve. One unit was defined as the amount of enzyme cleaving 1.0 nmol of the colorimetric substrate *p*NA per hour at 37°C under saturated substrate concentrations. The assays described above were repeated five times.

### Statistical Analysis

All experiments were performed in biological replicates. Data are presented as the mean ± SD. The statistical significance was determined by one-way ANOVA followed by Duncan’s multiple range tests to determine the differences in the mean values as compared with the controls. The criterion for significance of the difference between means was *p* < 0.05.

## Results

### Target Identification of miR-31

**Figure 1:**
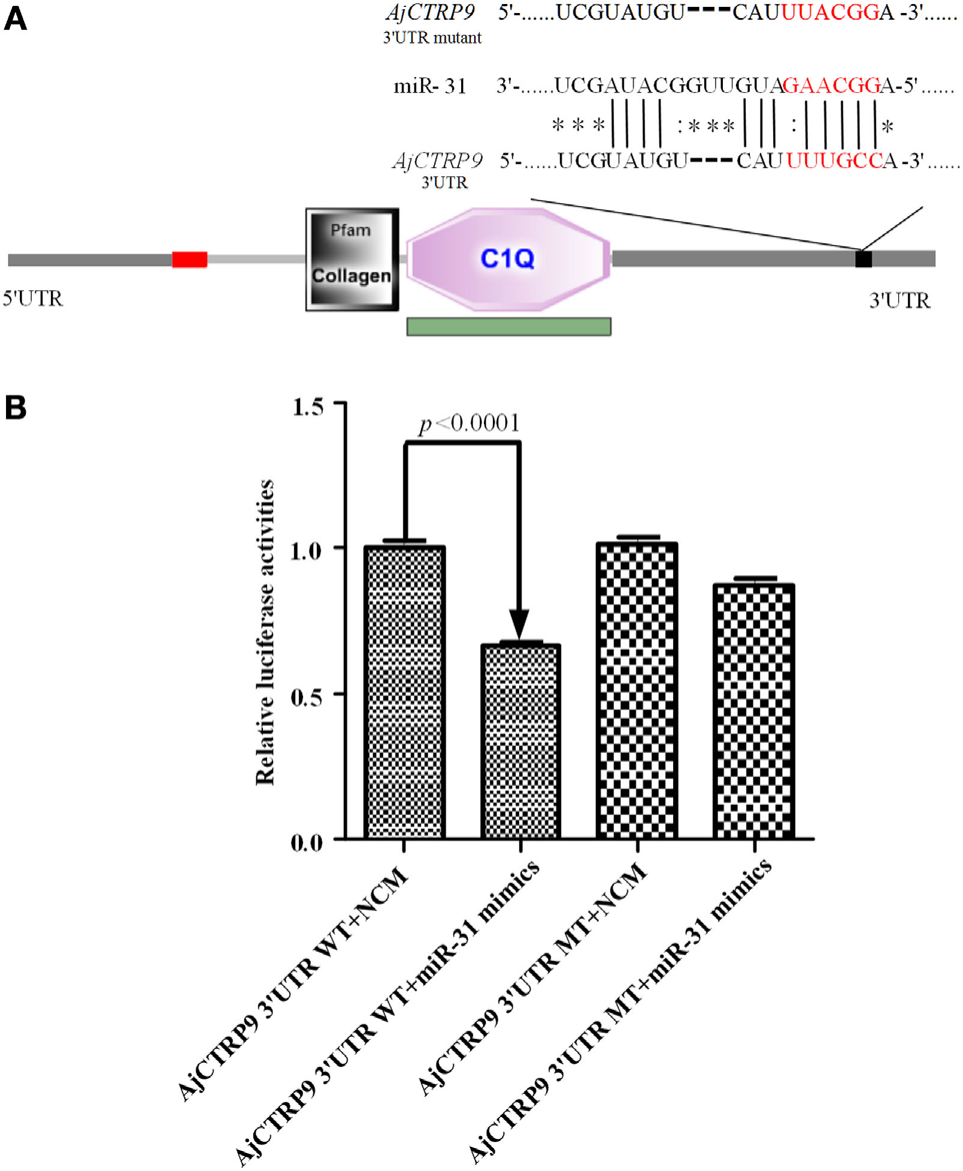
**Identification and characterization of the miR-31 binding sites in the 3’-untranslated region (3’-UTR) of *AjCTRP9***. **(A)** Schematic representation of putative miR-31 binding sites in the *AjCTRP9* 3’-UTR and the mutant sites (the protein domain of AjCTRP9 was annotated with SMART at http://smart.embl.de/), the red letters represent “seed” regions. **(B)** *AjCTRP9* luciferase reporter assays conducted in HEK-293T cells carrying the wild-type *AjCTRP9* 3’-UTR and a mutant *AjCTRP9* 3’-UTR (final concentration: 100 nM).

On the basis of the transcriptome data of sea cucumber *A. japonicus* (33, 34), the target of miR-31 was bioinformatically predicted by the miRanda v3.01 toolbox. Information on the binding sites of miR-31 in the 3’-UTR of *AjCTRP9* and the mutated sites is shown in Figure 1A. Imperfect pairing with a GU mismatch was allowed in many instances (43–45). To verify the prediction, we performed dual-luciferase reporter assays in HEK-293T cells transfected with miR-31 or control mimics together with the luciferase reporter plasmids containing either the *AjCTRP9* 3’-UTR WT or the *AjCTRP9* 3’-UTR MT (Figure 1B). Overexpression of miR-31, compared with the control mimics, significantly decreased the level of luciferase activity containing the entire 3’-UTR by 33.38% (*p* < 0.0001), and the results were not significantly different for transfection with the mutant vector, thus indicating that the *AjCTRP9* gene may be a target of miR-31.

### Cloning and Sequence Analysis of AjCTRP9

The full-length cDNA of *AjCTRP9* was generated by overlapping original fragments from ESTs and using a 3’-RACE approach in the sea cucumber *A. japonicus* and was deposited in GenBank under accession number KX50326. The cDNA sequence of *AjCTRP9* was 1,718 bp in length and contained a 909 bp ORF encoding a predicted product with 302 amino acid residues with a molecular weight of 32.37 kDa and a theoretical *pI* of 5.37 (Figure S1 in Supplementary Material). Smart analysis showed that the deduced amino acid sequence of AjCTRP9 contains a short N-terminal signal peptides (residues 1–23), a short variable region (residues 25–43), a collagen domain with 34 “Gly-X-Y” repeats (residues 45–155), and an a-terminal globular domain that is homologous to c1q domain immune complement component 1q (C1q) (residues 161–299) (Figure 1A; Figure S1 in Supplementary Material). There were a total of nine proline residues at the third position (Gly-X-Pro) within the collagen domain, which is often hydroxylated to enhance the stability of the collagen triple-helical structure (46). In addition, its collagen domain also had three conserved lysine residues that conformed to the consensus sequence (Gly-X-Lys), which can potentially be hydroxylated and glycosylated. Furthermore, multiple alignments indicated that AjCTRP9 shares 47.3% homology with *Saccoglossus kowalevskii* CTRP9 (XP_002739445.1), 44.0% homology with *Homo sapiens* CTRP9 (AAH40438.1), and 40.9% homology with *Danio rerio* CTRP9 (XP_005162770.1). Because a short N-terminal variable region was present in the CTRP9 from invertebrates and had the lowest similarity to CTRP9 from vertebrates, we speculated that the evolution of invertebrate CTRP9 may have been earlier than that of the other CTRP9 members (Figure S2 in Supplementary Material).

### Expression Analysis of AjCTRP9 and miR-31 in Immunity

**Figure 2:**
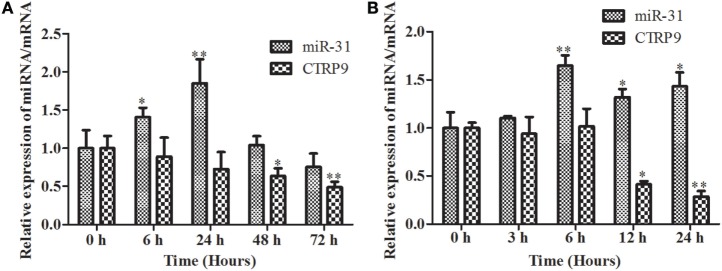
**The time course of expression patterns of miR-31 and *AjCTRP9***. **(A)** *AjCTRP9* mRNA transcripts and miR-31 expression in *Vibrio splendidus*-challenged *Apostichopus japonicus*. **(B)** *AjCTRP9* mRNA transcripts and miR-31 expression in *LPS*-treated coelomocytes. Values are given as the mean ± SD, *n* = 5. Asterisks indicate significant differences: *p < 0.05, **p < 0.01.

The temporal expression levels of miR-31 and *AjCTRP9* in coelomocytes post *V. splendidus* challenge and LPS treatment are shown in Figure 2. After the challenge with *V. splendidus*, the level of miR-31 significantly increased by 1.41-fold (*p* < 0.05) during the first 6 h, as compared with the control group and reached peak expression at 24 h with 1.85-fold (*p* < 0.01) increase (Figure 2A). Additionally, after exposure to 10 μg mL^−1^ LPS, the level of miR-31 sharply increased by 1.65-fold, as compared with the control (*p* < 0.01) during the first 6 h, and the elevated expression was maintained until 24 h (Figure 2B). However, the *AjCTRP9* transcript showed an opposite expression trend from that of miR-31 in response to both *V. splendidus* challenge and LPS treatment (Figure 2). The transcription of *AjCTRP9* was gradually downregulated and exhibited the least expression at 72 h, with a 0.49-fold (*p* < 0.01) decrease after the *V. splendidus* challenge (Figure 2A). The minimal expression level of *AjCTRP9* after the LPS stimulation was detected at 24 h, with a 0.29-fold (*p* < 0.01) decrease compared with the control group (Figure 2B).

### Loss- and Gain-of-Function Assay of miR-31

**Figure 3:**
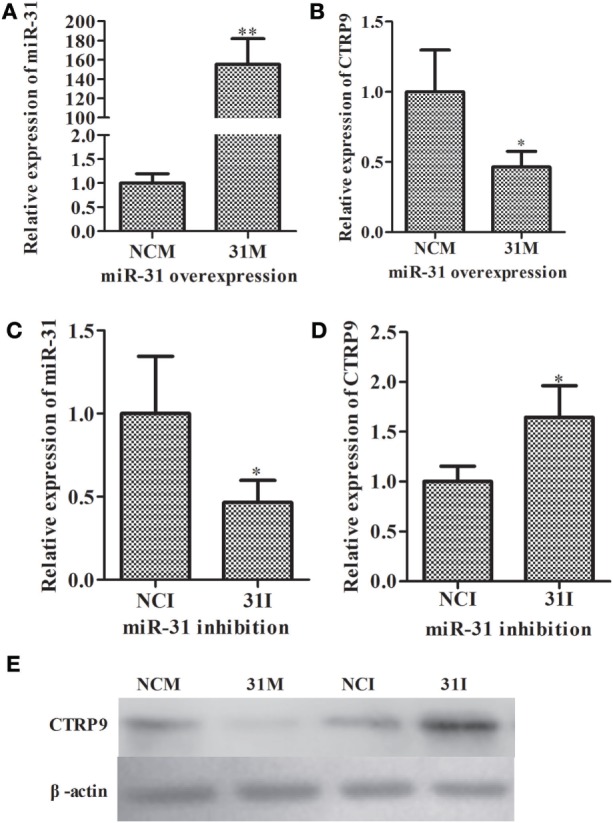
**Functional analysis of miR-31 and *AjCTRP9 in vivo***. **(A)** Relative miR-31 expression level after transfection with modified microRNA (miRNA) mimics. **(B)** Relative expression level of *AjCTRP9* after transfection with modified miRNA mimics. **(C)** Relative miR-31 expression level after transfection with modified miRNA inhibitor. **(D)** Relative expression level of *AjCTRP9* after transfection with modified miRNA mimics. **(E)** Western blot analysis of AjCTRPP protein after transfection with modified miRNA mimics or inhibitor. Values are given as the mean ± SD, *n* = 5. Asterisks indicate significant differences: *p < 0.05, **p < 0.01.

To further elucidate the functional roles of miR-31 in regulating *AjCTRP9*, loss-of-function or gain-of-function assays of miR-31 were performed *in vivo*. As shown in Figure 3, quantitative PCR indicated that the overexpression of miR-31 (Figure 3A) significantly decreased the mRNA expression of *AjCTRP9* by 0.46-fold (*p* < 0.05) (Figure 3B). Accordingly, the inhibition of miR-31 (Figure 3C) resulted in the elevated expression of *AjCTRP9* by 1.65-fold (*p* < 0.05) (Figure 3D). Furthermore, western blot analysis of the aberrant expression of miR-31 revealed that the protein abundance of AjCTRP*9* was decreased after miR-31 overexpression and increased after miR-31 inhibition (Figure 3E), in agreement with the *AjCTRP9* mRNA expression levels. Our results indicated that miR-31 directly targeted the *AjCTRP9* gene.

### Lipidomic Analyses of *A. japonicus* Coelomocytes after siRNA-Mediated AjCTRP9 Silencing

**Figure 4:**
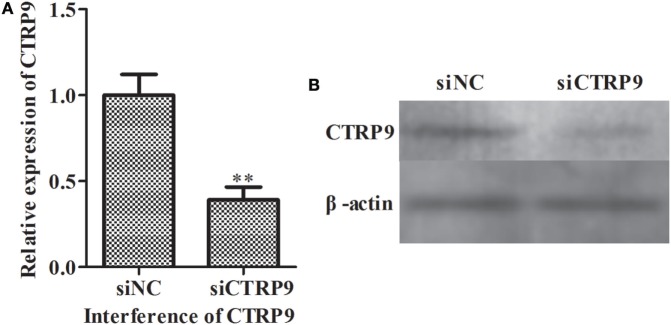
**The expression level of AjCTRP9 after transfection with specific small-interfering RNAs (siRNAs)**. **(A)** Relative expression level of *AjCTRP9* after *AjCTRP9* siRNA transfection. **(B)** Western blot analysis of AjCTRPP protein after *AjCTRP9* siRNA transfection. Values are given as the mean ± SD, *n* = 5. Asterisks indicate significant differences: *p < 0.05, **p < 0.01.

From the above results, we observed that miR-31 directly targets the *AjCTRP9* gene. As a CTRP family member, CTRP9 plays important roles in multiple physiological processes, especially in fatty acid oxidation (47). To further investigate the possible biological roles of the *AjCTRP9*, the lipidomics of coelomocytes were analyzed by LC-MS after *AjCTRP9* silencing. Figure 4 shows the expression levels of *AjCTRP9* after specific siRNA transfection. Our results showed that the injection of *AjCTRP*9 siRNA specifically inhibited *AjCTRP9* transcript expression (Figure 4A) and protein expression (Figure 4B) *in vivo*. Therefore, the changes in the lipid metabolites of coelomocytes were identified in the siAjCTRP9 and NC groups.

**Figure 5:**
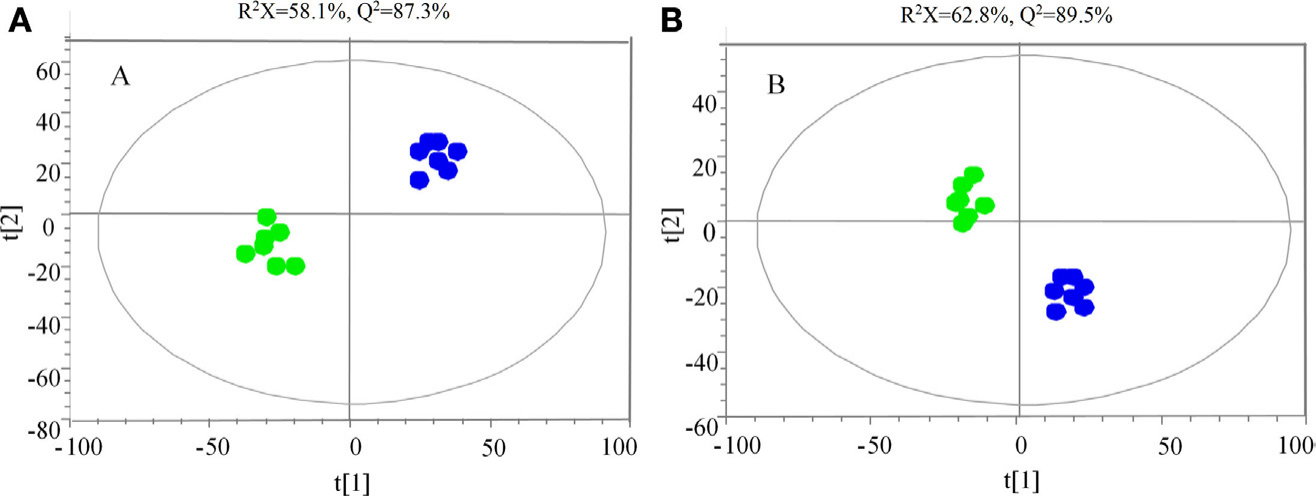
**Principal component analysis score scatter plot in ESI^+^ (A) and ESI^−^ (B) mode for the total lipids of *Apostichopus japonicus* between the siAjCTRP9 group (green) and the negative control group (blue)**.

**Figure 6:**
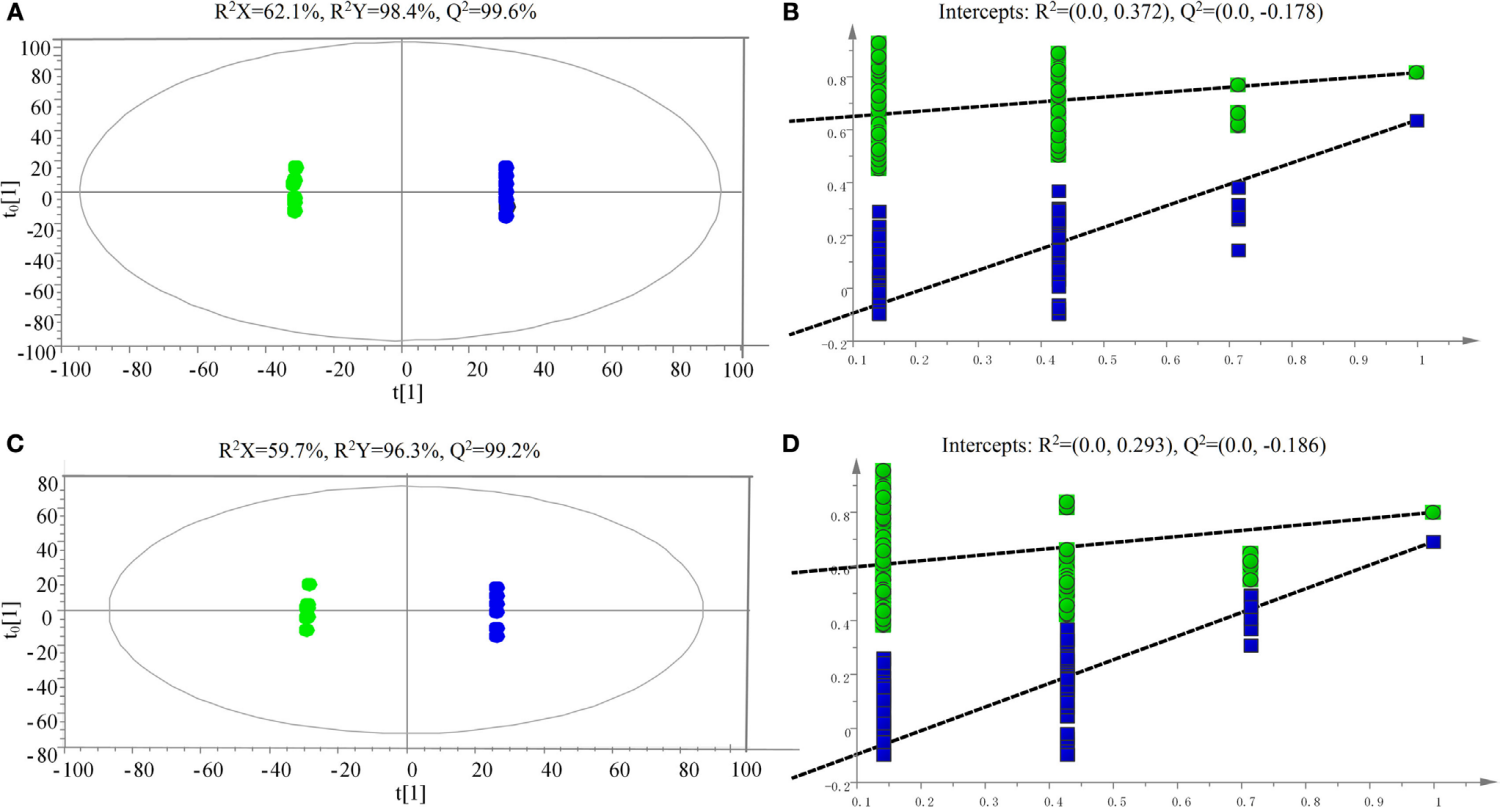
**Scatter plot of OPLS-DA scores (A,C) and validation plot (B,D) of the PLS-DA analysis on siAjCTRP9 group in both positive (A,B) and negative (C,D) ion scan modes compared with the negative control group**.

**Figure 7:**
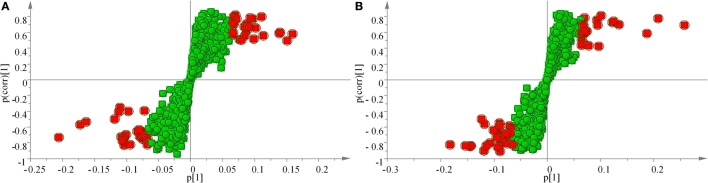
**OPLS loading S-plot for the total lipids of the siAjCTRP9 group and the negative control group detected in the ESI^+^ (A) and ESI^−^ (B) modes**. Significant ions are outlined in red and listed in Table 1.

The samples were characterized by LC-MS in both positive and negative ion mode. The mass spectral data were processed by multivariate analysis. The PCA score plots (Figure 5) in both ion modes showed that the two samples of *A. japonicus* were clearly separated by the first components. A trend of time-dependent cluster formation by the first two components was observed in both ESI^+^ and ESI^−^ modes, thus explaining 58.1% (Figure 5A) and 62.8% (Figure 5B) of the all variances, respectively. The OPLS-DA score plot showed significant lipidomic differences between the siAjCTRP9 and NC groups (Figure 6). As shown in the positive ion mode (Figure 6A), the parameters *R*^2^*X, R*^2^*Y*, and *Q*^2^ revealed the high discriminative and predictive ability of the model. Further model validations in PLS-DA, with the number of permutations equal to 200, generated intercepts of *R*^2^ = 0.372 and *Q*^2^ = −0.178 (Figure 6B). Along with those obtained in negative mode (Figures 6C,D), these results demonstrated that the OPLS-DA models were well fitting and statistically valid. The S-plot visualizes the covariance and correlation among the metabolites, and thus can be used to identify important metabolites (48). The significant metabolites at the top and bottom of the S-plots (Figure 7) were related to the group separation and were selected as potential biomarkers (Table 1) according to parameters of variable importance in the projection (VIP). The relative abundance of significant ions that were higher in the siAjCTRP9 group compared with the NC group in sea cucumber were located in the upper-right quadrant (with >0 of correlation) of the S-plot after the OPLS-DA approach (Figure 7), including seven phosphatidylcholines (PC) (18:1/18:2; 18:2/17:1; 18:1/18:1; 15:0/18:2; 20:1/18:2; 15:0/20:2; 18:2/18:2), three Cers (d15:1/20:0; d15:1/21:0; d17:1/23:1), and TAG (16:0/16:0/16:0), whereas those that were lower in abundance were in the lower-left quadrant (with <0 of correlation), including four PC (17:1/20:5; 17:1/20:4; 15:0/20:5; 22:6/13:0), two phosphatidylethanolamine (PE) (17:1/20:4; 18:1/20:5), two lysophosphatidylcholine (LPE) (20:5; 20:4), three TAG (18:1/18:1/16:0; 18:1/18:1/16:1; 18:1/16:0/20:2), and (LPC) (18:2).

**Table 1:** **Summary of the metabolite ions of *Apostichopus japonicus* that showed significant changes between the siAjCTRP9 and negative control groups**.

RT (min)	*m*/*z*	Annotation	Cov	Cor	Variable importance in the projection	Fold changes
8.17	798.5	[M + Li]^+^ 17:1/20:5-PC	−0.205713	−0.732526	8.16913	0.72
8.51	784.5	[M + H]^+^ 18:1/18:2-PC	0.160094	0.578532	6.35124	3.66
8.99	770.5	[M + H]^+^ 18:2/17:1-PC	0.151788	0.490732	6.02605	1.42
9.41	786.5	[M + H]^+^ 18:1/18:1-PC	0.139849	0.59799	5.54959	3.98
8.97	800.5	[M + Li]^+^ 17:1/20:4-PC	−0.117883	−0.503678	4.67847	0.76
8.83	744.5	[M + H]^+^ 15:0/18:2-PC	0.113798	0.558261	4.51796	1.79
10.65	842.5	Unknown	−0.106533	−0.774079	4.22787	0.68
8.01	772.5	[M + Li]^+^ 15:0/20:5-PC	−0.103263	−0.735489	4.09839	0.67
9.96	558.5	[M + Li]^+^ d15:1/20:0-Cer	0.0973946	0.768276	3.86752	3.68
10.66	572.5	[M + Li]^+^ d15:1/21:0-Cer	0.0937542	0.782561	3.72185	4.97
1.92	464.3	[M-H]^−^ 20:5-LPE	−0.122912	−0.502297	3.64418	0.76
9.61	812.5	[M + H]^+^ 20:1/18:2-PC	0.0910315	0.669488	3.61434	5.20
12.08	626.5	[M + Li]^+^ d17:1/23:1-Cer	0.0880302	0.706791	3.49483	1.22
3.87	339.2	Unknown	−0.116787	−0.562221	3.47243	0.75
10.04	772.5	[M + H]^+^ 15:0/20:2-PC	0.0796566	0.502002	3.15563	4.21
1.85	466.3	[M-H]^−^ 20:4-LPE	−0.106769	−0.801495	3.14981	0.55
18.64	865.7	[M + Li]^+^ 18:1/18:1/16:0-TAG	−0.0676901	−0.780218	2.68574	0.32
18.1	863.7	[M + Li]^+^ 18:1/18:1/16:1-TAG	−0.0673214	−0.824131	2.67018	0.22
7.14	770.5	[M + Li]^+^ 22:6/13:0-PC	−0.0659779	−0.799264	2.61918	0.75
7.7	782.5	[M + H]^+^ 18:2/18:2-PC	0.065864	0.56148	2.6143	2.76
18.56	813.7	[M + Li]^+^ 16:0/16:0/16:0-TAG	0.0621351	0.674626	2.4658	2.23
11.27	802.5	Unknown	−0.0613742	−0.530681	2.43706	0.80
18.7	891.7	[M + Li]^+^ 18:1/16:0/20:2-TAG	−0.0608799	−0.805357	2.41512	0.23
1.37	492.3	[M-CH_3_]^−^ 18:2-LPC	−0.0555527	−0.560026	1.6503	0.58
10.33	750.5	[M-H]^−^ 17:1/20:4-PE	−0.0552554	−0.363959	1.63801	0.96
10.08	762.5	[M-H]^−^ 18:1/20:5-PE	−0.0460899	−0.551591	1.37487	0.66

*Ion ranks from the loading S-plot of OPLS-DA analysis showed the rank of ions with the highest confidence and greatest contribution to the separation between the siCTRP9 and NC groups. The ion rank is synonymous to labeling the ions with relevant *m*/*z* as in Figure 7. Cov. (covariance), Cor. (correlation), and VIP were obtained from the OPLS-DA analysis by using the datasets from positive or negative ion scan modes to determine the ESI^+^ and ESI^−^ corresponding to the positive and negative ions of the relevant adducts, respectively. Fold change equals the peak area observed in the siAjCTRP9 group compared with the NC group*.

### AjCTRP9 Knockdown Induces Accumulation of Cer Mass Mainly through Sphingosine Metabolism

**Figure 8:**
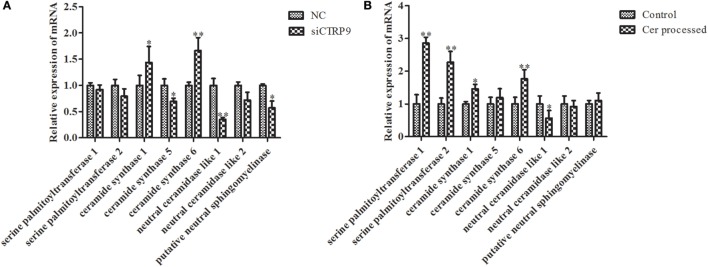
**The mRNA expression levels of relative genes after *AjCTRP9* silencing (A) and Cer treatment (B)**. Values are given as the mean ± SD, *n* = 5. Asterisks indicate significant differences: *p < 0.05, **p < 0.01.

According to significantly different metabolites (Table 1), we found that the three identified Cer species (d15:1/20:0; d15:1/21:0; d17:1/23:1) were dramatically increased by 3.68-, 4.97-, and 1.22-fold after *AjCTRP9* interference, respectively. Cer is a condensation product of the amino alcohol sphingosine and a fatty acid during an acylation reaction. It is a key intermediate in sphingolipid metabolism and an important signaling molecule in determining cell fate (49). To understand which biological process resulted in the Cer accumulation after *AjCTRP9* deletion, we investigated the fluctuations in expression of Cer metabolism-related genes by qPCR (Figure 8A). As shown in Figure 8A, the expression levels of *Cer synthase 1* and *Cer synthase 6* were significantly increased by 1.44-fold (*p* < 0.05) and 1.66-fold (*p* < 0.01) after *AjCTRP9* silencing, respectively, whereas the *Cer synthase 5* transcript was downregulated by 0.69-fold (*p* < 0.05). In addition, the mRNA levels of *neutral ceramidase 1* and *putative neutral sphingomyelinase* both sharply decreased by 0.35-fold (*p* < 0.01) and 0.57-fold (*p* < 0.05), respectively, after interference. There were no significant changes in *serine palmitoyltransferase (SPT) 1* and *SPT 2* transcripts after *AjCTRP9* knockdown. These results revealed that the increase in the three Cer contents might have represented the *Cer synthases* after silencing of *AjCTRP9*, in which different *Cer synthases N*-acylated sphingosine to form the different Cers.

### C_6_ Cer Exposure Increases SPT mRNA Expression and Directly Promotes Cell Apoptosis

**Figure 9:**
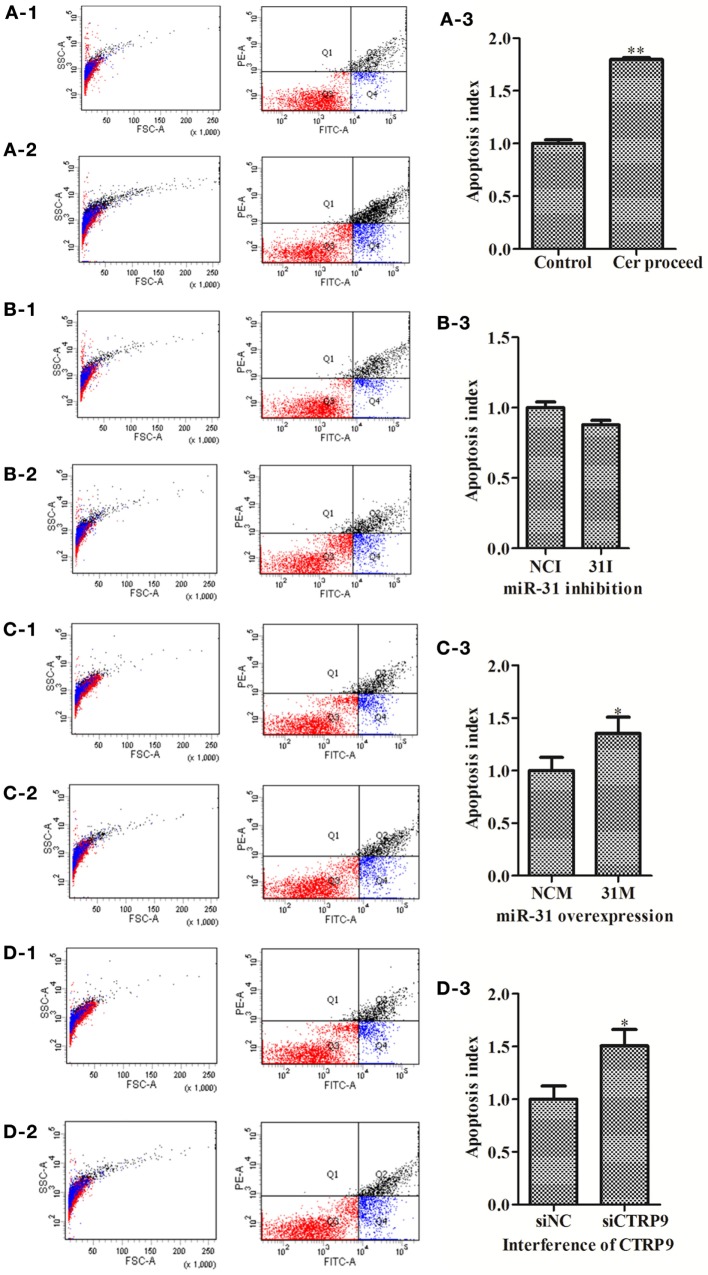
**The apoptosis rate of *Apostichopus japonicus* coelomocytes**. **(A-1–A-3)** Cer treatment; **(B-1–B-3)** miR-31 inhibition; **(C-1–C-3)** miR-31 overexpression; **(D-1–D-3)** *AjCTRP9* silencing. Values are given as the mean ± SD, *n* = 5. Asterisks indicate significant differences: *p < 0.05, **p < 0.01.

To explore the functional effect of Cer on cell survival, we measured cell apoptosis after Cer treatment of *A. japonicus in vivo* (Figure 9). In response to the stimulation with 100 μg mL^−1^ Cer, the apoptosis rate was sharply increased by 1.80-fold (*p* < 0.01) compared with the control (Figures 9A1–A3), thus showing that the disturbance in Cer signaling directly induced cell death in *A. japonicus*. In addition, we found that the mRNA expression profiles of *SPT 1, SPT 2, Cer synthase 1*, and *Cer synthase 6* were upregulated by 2.86-fold (*p* < 0.01), 2.28-fold (*p* < 0.01), 1.46-fold (*p* < 0.05), and 1.77-fold (*p* < 0.05), respectively, after the exposure to exogenous Cer (Figure 8B). In contrast, the *neutral ceramidase* 1 transcript was decreased by 0.56-fold (*p* < 0.05) compared with the control, thus indicating that the overexpression of short Cer might lead to the enrichment of longer Cers in *A. japonicus* cells through a *de novo* synthesis pathway by *SPT* and that the exposure of exogenous Cer disturbs the sphingolipid metabolism in sea cucumber.

### miR-31 Regulates Apoptosis in *A. japonicus* Coelomocytes by Targeting AjCTRP9

To test the hypothesis that miR-31 regulates apoptosis in *A. japonicus* by targeting *AjCTRP9*, we further investigated the apoptosis of cells transfected with a series of reagents *in vivo* (Figure 9). Transfection with miR-31 mimics significantly induced cell apoptosis (1.35-fold, *p* < 0.05) 24 h post infection (Figures 9C1–C3). Downregulation of *AjCTRP9* with specific siRNA also induced apoptosis (1.51-fold, *p* < 0.05) under the same conditions (Figures 9D1–D3).

### miR-31 Targeting AjCTRP9 Modulates Cer Content and Induces Apoptosis through Caspase Cascades

**Figure 10:**
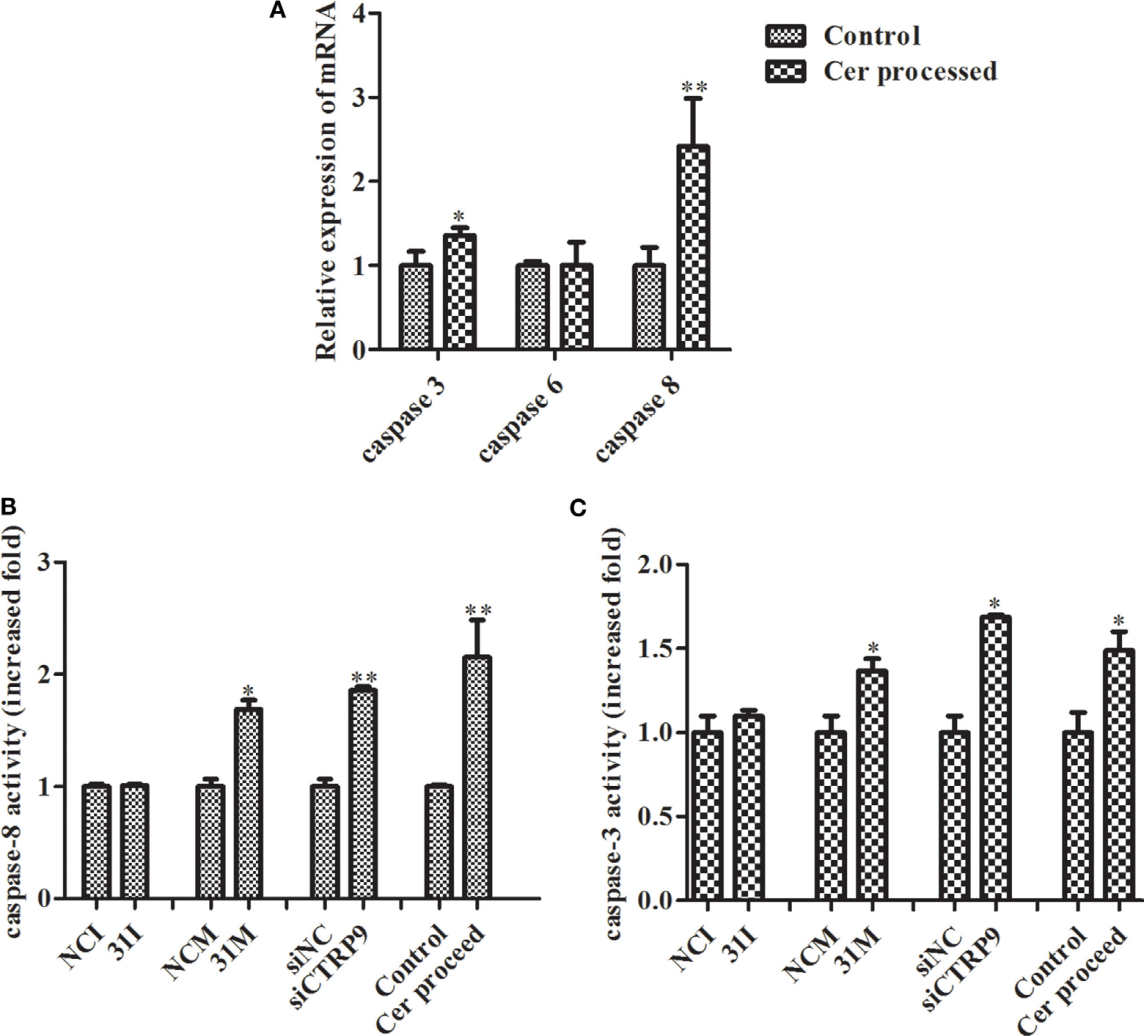
**Effects of Cer treatment on caspase activity in the coelomocytes of *Apostichopus japonicus***. **(A)** The mRNA expression level of caspase-3/-6/-8; **(B)** caspase-8 activity; **(C)** caspase-3 activity. Values are given as the mean ± SD, *n* = 5. Asterisks indicate significant differences: *p < 0.05, **p < 0.01.

**Figure 11:**
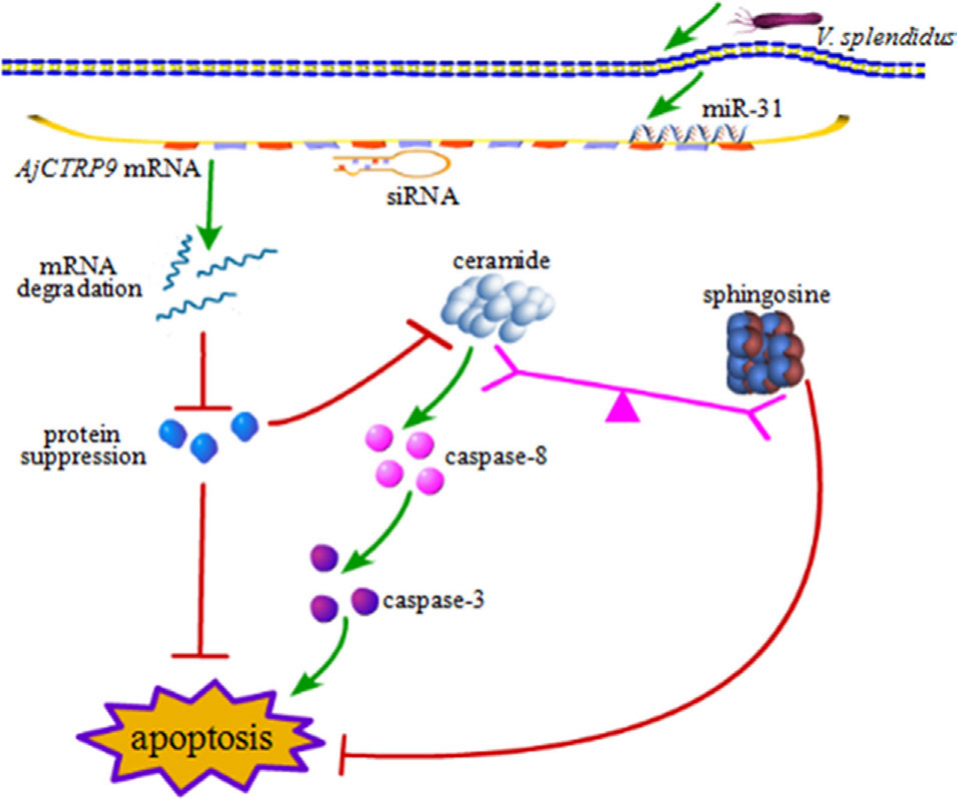
**Schematic representation of the involvement of miR-31 in disturbing the lipid metabolism balance by targeting *AjCTRP9***.

From the results above, we concluded that miR-31 regulates apoptosis by targeting *AjCTRP9* in *A. japonicus* through the overproduction of Cer. To reveal the molecular cascade of apoptosis regulated by signaling of Cer channels, we first examined the expression of apoptotic genes after Cer injection (50) (Figure 10A). We noticed that *caspase-3* and *caspase-8* transcripts were both markedly upregulated by 1.35-fold (*p* < 0.05) and 2.42-fold (*p* < 0.01), respectively, compared with the control, thus indicating that these two molecules play key roles in Cer-induced apoptosis. To accurately evaluate the caspase cascades, the enzymatic activities of caspases were measured from the *coelomocytes* after transfection with a series of reagents *in vivo* (Figures 10B,C). As shown in Figure 10B, caspase-8 activity was significantly induced after miR-31 overexpression, *AjCTRP9* silencing, and Cer exposure. The caspase-3 activity was also consistent with caspase-8 activity in every condition and had a greater magnitude after *AjCTRP9* knockdown, thus suggesting that caspase-8 directly modulates caspase-3 and promotes apoptosis. The regulatory interplay among miR-31, *AjCTRP9*, Cer, and apoptosis is diagrammed in Figure 11.

## Discussion

microRNAs are powerful regulators of gene expression under normal physiological processes or in pathological states (51). In our previous work, we have revealed that miR-31 modulates respiratory burst by targeting NF-κB (*p*105) during pathological development in sea cucumbers (52). Currently, accumulating evidence suggests that miRNAs are key players in the regulation of inflammation and metabolism (16, 53). A given miRNA may have multiple mRNA targets that are involved in nearly every biological process (54). miR-31 belongs to a highly evolutionarily conserved miRNA “seed family” that participates in the immune response, and whose abnormal expression or function in the immune system has been linked to multiple diseases (55–57). However, the limitations of these studies restrict the extrapolation to effects on immune defense and cell homeostasis after host–pathogen interactions. In the current study, we discovered a novel role of miR-31 in promoting cell apoptosis in *A. japonicus* by targeting *AjCTRP9*, which encodes an important adipokine involved in multiple physiological processes. The most salient finding of this paper is the identification of the role of miR-31 in regulating lipid-mediated signal transduction events during the interactions between bacterial pathogens and host cells.

Metabolism and immunity are often liked by proteins of dual function. CTRP9, a member of the CTRP family, has attracted much interest because of its anti-inflammatory and metabolic effects, especially in glucose metabolism and fatty acid oxidation (46, 58). In the present study, we found that the mRNA level of *AjCTRP9* was significantly decreased and showed opposite expression trends from those of miR-31 after *V. splendidus* challenge and LPS exposure in primary coelomocytes (Figure 2). Moreover, our results showed that the overexpression of miR-31 or silencing of *AjCTRP9 in vivo* resulted in an increase in the apoptotic rates (Figure 9), thus suggesting that miR-31 negatively targets *AjCTRP9* and induces cell apoptosis during the immune response. However, the apoptosis rate was not significantly changed after transfection with the miR-31 inhibitor (Figures 9B1–B3). In humans, CTRP9 levels are negatively associated with the amount of visceral fat and are positively associated with a favorable glucose or metabolic phenotype (31, 59). When overexpressed, CTRP9 significantly ameliorates palmitate- or tunicamycin-induced hepatic steatosis and apoptosis (60). Additionally, CTRP9 protects against acute cardiac damage in response to pathological stimuli by suppressing inflammatory reactions through adiponectin receptors (61). However, targeted deletion of CTRP9 increases food intake, decreases insulin sensitivity, and promotes hepatic steatosis in mice (58). These studies have clearly demonstrated that CTRP9 plays pivotal roles in modulating metabolism during inflammation. More importantly, the complementary gain-of-function and loss-of-function results support a role for CTRP9 in regulating lipid metabolism. In addition, our previous data obtained from high-throughput sequencing have revealed that the *AjCTRP9* transcript is sharply downregulated after miR-31 overexpression, and significant metabolic differences are also present in sea cucumbers with SUS (29, 30). On the basis of these results, we speculate that the abnormal expression of miR-31 targeting the *AjCTRP9*-induced apoptosis might perturb the lipid balance in the cells and may have contributed to SUS outbreaks in *A. japonicus*.

To explore the miR-31-mediated apoptosis resulting from perturbed cell lipid metabolism, we further investigated the lipidomics of coelomocytes analyzed by LC-MS after *AjCTRP9* silencing. The results indicated remarkable differences in lipid profiles of the two groups (Figures 5–7), and some lipid molecules including PC, LPC, TAG, PE, LPE, and Cer were significantly increased or decreased after *AjCTRP9* knockdown (Table 1). These lipids have previously been shown to have multiple critical roles in cellular functions, such as cell growth, signal transduction, membrane homeostasis, and apoptosis (5, 62, 63). From these results, we concluded that the miR-31-targeted modulation of *AjCTRP9* expression may be an extremely important mechanism in lipid metabolism. Notably, three types of identified Cers (d15:1/20:0; d15:1/21:0; d17:1/23:1) were synchronously increased after *AjCTRP9* interference, thus indicating that the Cer species might be promising biomarkers for lipid metabolism imbalance. Cers are important intermediators of sphingolipid metabolism and function not only as structural components of cell membranes but also as key mediators of apoptosis triggered by various stimuli in most cells (64, 65). Accumulating evidence indicates that excessive levels of Cer directly promote cell apoptosis (66, 67). In this study, we also observed that the apoptosis rate was significantly increased after Cer exposure (Figure 9). Hence, we confirmed that the downregulation of *AjCTRP9* by miR-31 mainly results in the production of Cers and further induces apoptosis in coelomocytes. Cers are mainly produced from the *de novo synthesis* pathway regulated by the enzyme *SPT* (68, 69). Alternatively, they can also be generated by the sphingosine salvage pathway through the action of *Cer synthases* or the reverse activity of *neutral ceramidase* (70). Ogretmen et al. have determined that exogenous short-chain C_6_ Cer induces the production of endogenous long-chain Cer in the A549 human adenocarcinoma cell line through the recycling of the sphingosine backbone (71). Grether-Beck et al. have reported that stimulation of keratinocytes with exogenous C_6_ Cer induces *de novo* Cer synthesis in human keratinocytes through the induction and activation of the *SPT* mRNA (72). In our case, there were no significant changes in the *SPT* transcript after silencing *AjCTRP9*, but its mRNA expression was markedly increased after Cer injection (Figure 8), thus indicating that treatment with short Cers might result in the synthesis of longer Cers. In addition, sphingomyelin or sphingosine hydrolysis in cell membranes also contributes to the production of Cers by different metabolic enzymes (69). We found that the mRNA levels of *Cer synthase 1* and *Cer synthase 6* were both notably upregulated, whereas *neutral ceramidase 1* and *putative neutral sphingomyelinase* transcripts were both significantly downregulated after *AjCTRP9* knockdown. Clearly, the overproduction of Cers after *AjCTRP9* interference occurs mainly as a result of sphingosine hydrolysis. Moreover, *Cer synthase 1, Cer synthase 6*, and *neutral ceramidase 1* transcript levels after Cer challenge displayed almost identical expression profiles with the levels after silencing *AjCTRP9*. A study by Osawa et al. has indicated that the dynamic balance between the intracellular levels of Cers and sphingosine may determine cell survival (73). Hence, the aberrant expressions of metabolic enzymes regulate Cer channel formation and induce cell apoptosis. Our present findings indicated that miR-31 negatively modulates the expression of *AjCTRP9* and the disturbance in Cer channels, thereby initiating apoptosis. Apoptosis is a very complex multistep process dependent on several apoptosis-related molecules, but its signaling is triggered by either the extrinsic pathway or the intrinsic pathway (74). The primary regulators of apoptosis are a family of cysteine-aspartic specific proteases known as caspases (75). Our results showed that the levels of *caspase-3* and *caspase-8* mRNAs were significantly induced after Cer treatment (Figure 10A). Furthermore, the enzymatic activities of the initiator caspase-8 and the executioner caspase-3 were both clearly upregulated after miR-31 overexpression and *AjCTRP9* silencing, as well as exogenous Cer exposure (Figures 10B,C), thus suggesting that the sequential activation of caspases plays critical roles in the final steps of apoptosis.

In summary, the present study provides novel evidence that miR-31 plays a key role in Cer channel formation by targeting *AjCTRP9*, thereby linking immune defense with lipid metabolism (Figure 11). miR-31, a highly conserved and multifunctional regulator that negatively targets *AjCTRP9* and disturbs the lipid metabolism balance, further results in Cer channel formation, thus ultimately leading to cell apoptosis during host–pathogen interactions. These new findings may provide new therapeutic perspectives with which to suppress miR-31 expression in sea cucumbers with SUS.

## Data Sharing

Requests for access to the data, statistical code, questionnaires, and technical processes may be made by contacting the corresponding author at lichenghua@nbu.edu.cn.

## Ethics Statement

The sea cucumbers (*A. japonicu*s) used here were commercially cultured, and all the experiments were conducted in accordance with the recommendations in the Guide for the Care and Use of Laboratory Animals of the National Institutes of Health. The study protocol was approved by the Experimental Animal Ethics Committee of Ningbo University, China.

## Author Contributions

CL and YS conceived and designed the experiments; YS, PZ, and XZ performed the experiments; YS, CL, and WZ analyzed the data; CL and WZ contributed reagents/materials/analysis tools; YS, CL, and WX wrote the paper.

## Conflict of Interest Statement

The authors declare that the research was conducted in the absence of any commercial or financial relationships that could be construed as a potential conflict of interest.

## Footnotes

^1^http://www.ncbi.nlm.nih.gov/blast.

^2^http://www.expasy.org/.

^3^http://smart.embl-heidelberg.de/.

^4^http://www.expasy.ch/tools/protparam.html.

^5^http://www.ebi.ac.uk/clustalw/.
