# Supplementary material for: miR-31 Links Lipid Metabolism and Cell Apoptosis in Bacteria-Challenged Apostichopus japonicus via Targeting CTRP9
Source: Front Immunol. 2017 Mar 13;8:263. doi: 10.3389/fimmu.2017.00263 (PMC5346533; doi:10.3389/fimmu.2017.00263)
Supplement: Supplementary file 1 [file Image_1.PDF]

Figure S1

1 TATTTCCAGAATAAAGCCACAAGTTGTACACAGTGGTAATCCTGGGCTGGCAGCCAAGAA
61 CGAGACCAGCATCAAAACAAGTCCAGCTACAGTACTAGAAACATCATCACCTTTCCATGC
1 M D P F K E H L P Q I F L A A L F
121 AGAGAGAAC**ATG**GATCCGTTTAAAGAGCATCTACCTCAAATATTCTTGGCAGCTCTTTTC
18 I L G A K A N N M D D T L S L P E G V N
181 ATCCTTGGAGCAAAGGCTAACAATATGGATGACACTTTATCACTGCCAGAGGGTGTTAAT
38 D R N T Q Q C A C C S R G D P G P Q G M
241 GACAGGAACACACAGCAATGTGCATGTTGTTCACGAGGAGACCCTGGCCCTCAGGGTATG
58 T G L P G I Q G L P G V P G S P G S H G
301 ACTGGCTTACCTGGAATACAGGGCCTCCCAGGGGTACCAGGATCTCCTGGTAGTCATGGA
78 T N G I P G L H G N K G D L G N P G M V
361 ACTAACGGCATTCCAGGATTACATGGTAACAAAGGTGACCTTGGAAACCCAGGAATGGTG
98 G P E G P Q G E P G L L G L P G K I G L
421 GGTCCAGAAGGACCCCAAGGGGAACCGGGCCTCCTAGGACTTCCAGGAAAGATTGGCCTC
118 Q G S R G P R G H S G P T G A E G P G G
481 CAAGGATCGAGAGGACCGAGAGGTCATTCAGGCCCGACTGGGGCAGAAGGACCAGGCGGT
138 P R G I K G D K G E R G S S S S W G G L
541 CCCAGAGGCATAAAGGGGGATAAGGGAGAAAGGGGATCGTCTAGCTCTTGGGGGGGCTTG
158 R Y G P Y H V H S Q S A F S V A S S K E
601 CGGTATGGACCCTATCACGTGCACAGCCAGTCGGCATTCTCTGTGGCGTCCAGTAAAGAG
178 I Q A E P I E D T I L I F D T I F V N I
661 ATCCAGGCAGAACCCATAGAGGACACCATACTAATCTTCGATACCATATTTGTCAATATC
198 G N D F D V A H G V F H C R I N G T Y Y
721 GGCAACGACTTTGACGTAGCGCACGGTGTCTTCCACTGCCGGATAAACGGCACCTATTAC
218 F I I H A N K W S N Q N D L Y L K L M K
781 TTCATTATACATGCAAACAAATGGTCCAATCAGAACGACCTATACCTGAAGCTGATGAAG
238 N D V M V I G L Y E D A G Y D Y Y D M T
841 AATGACGTGATGGTGATAGGACTGTACGAAGACGCGGGATACGACTATTACGATATGACC
258 S N S I M L H L V E E D Q V W L Q L H I
901 AGTAACAGCATCATGTTACATCTAGTTGAGGAGGACCAGGTCTGGCTACAACTCCATATT
278 N N R V Y G G S S R M T T F S G W M I Y
961 AATAACAGGGTGTACGGGGGAAGCTCCAGGATGACTACCTTCTCTGGCTGGATGATATAT
298 E D P I P *
1021 GAGGATCCTATACCC**TGA**GAGGAAGACTAAACCAGTCCAGAAATGGGTGTCGCA***ATTTA***G
1081 CAGAGAGATTTGGTGGAAAATATTTGGTAAAAACATAGGTAGTGAAGTGTATGTACAAGT
1141 AATGGAAGGTTTTATAAAGGGAGCGAGCAGGGAAAGGTACTTTTAACG***ATTTA***GTACACT
1201 TGCAAACCAAGTGTCTGATGTTTACAGATATCACTTTGAAGTGGTAGCACGGTACGATTT
1261 TCCATTGACGGGTGTGGCATTGATCATTTCACTTTGAAGTGGTAACACGGTTAGGGTTTT
1321 TCATTGAGGGGTATGGCATTGATCGCATCAAAAGAAAATATCCGGCAATTACTGCCGATG
1381 CAAATAGCATTTGGAGAGAGAAAACAATATGGATCTGTATCAGATCATCTGTATATTAAT
1441 TGGTAGCAGTGGATAAACTTCAATAGAGAAGCCCTATATAATCTGAAGCAAAATACAAAA
1501 AGGAAACTAAGAATCGAGAGCAGATCGTATGTCAT**TTTGCC**AATTGTAATTGCTCTGTTG
1561 AACACAATTAATATTCAACAGAGAGATGTGAAGGATTCCTTAAAAGAGTTCTAAAGTCAA

1621 AGATCTGAGCAAATTTTCCAAGAGCTATGAAATGGCCAC***ATTTA***TATGTAAATTATAGAC
1681 C***AATAAA***GTAACTATTTTTGTGGGACACAAAAAAAAAA

Supplementary Figure 1 | The complete nucleotide and deduced amino acid sequence of *Apostichopus japonicus* CTRP9. The start codon was blacked. The asterisk indicated the stop codon. The signal peptides were shown in gray shadow, a variable region was underlined by broken-lines, a collagen domain was underlined, and the c1q domain was double underlined. The bingding sites of miR-31 was bold. The RNA instability sequences (ATTTA) and polyadenylation signal (AATAAA) were indicated by bold italics.
