# Supplementary material for: miR-31 Links Lipid Metabolism and Cell Apoptosis in Bacteria-Challenged Apostichopus japonicus via Targeting CTRP9
Source: Front Immunol. 2017 Mar 13;8:263. doi: 10.3389/fimmu.2017.00263 (PMC5346533; doi:10.3389/fimmu.2017.00263)
Supplement: Supplementary file 2 [file Image_2.PDF]

Figure S2

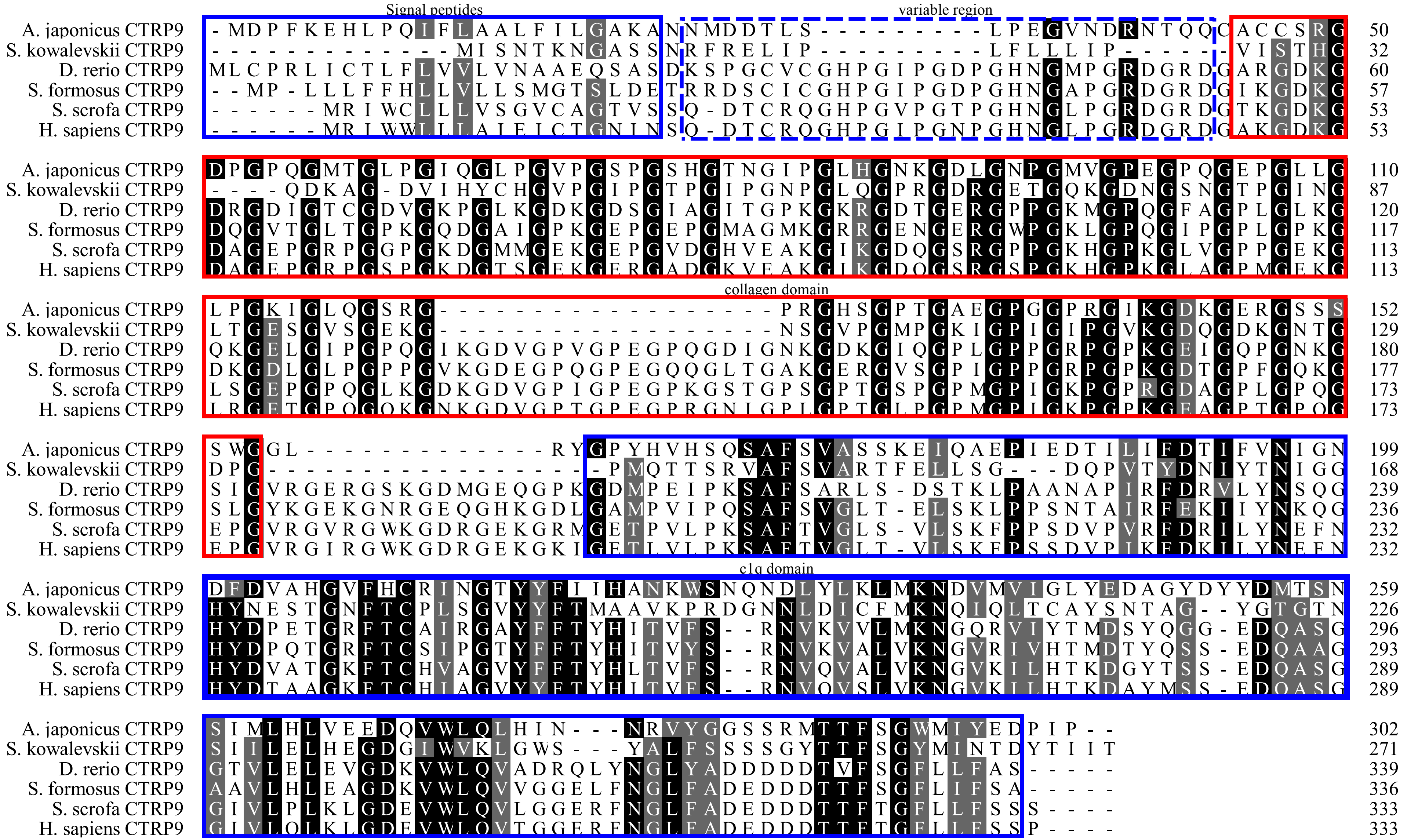
Signal peptides
variable region
collagen domain
c1q domain
A. japonicus CTRP9
S. kowalevskii CTRP9
D. rerio CTRP9
S. formosus CTRP9
S. scrofa CTRP9
H. sapiens CTRP9

Supplementary Figure 2 | Alignment of the predicted amino acid sequences of CTRP9 using ClustalW2 Multiple Alignment program. The consensus residues were shaded with a threshold of more than 80% identity using Multiple Align Show program. Identical residues were indicated in black, and similar residues in light gray. Domains of CTRP9 proteins were predicted by SMART program and high lighted by solid-lined boxes, including signal peptides, collagen domain, and c1q domain. The variable region was high lighted by broken-lines boxes. The accession numbers of CTRP9 proteins from GenBank were as follows: *Homo species* CTRP9 (AAH40438.1), *Sus scrofa* CTRP9 (XP_003130932.1), *Danio rerio* CTRP9 (XP_005162770.1), *Scleropages formosus* CTRP9 (KPP60699.1), and *Saccoglossus kowalevskii* CTRP9 (XP_002739445.1), respectively.
