# Supplementary material for: miR-31 Links Lipid Metabolism and Cell Apoptosis in Bacteria-Challenged Apostichopus japonicus via Targeting CTRP9
Source: Front Immunol. 2017 Mar 13;8:263. doi: 10.3389/fimmu.2017.00263 (PMC5346533; doi:10.3389/fimmu.2017.00263)
Supplement: Supplementary file 3 [file Table_1.DOCX]

**Table S1.** Summary of primers used in this study. The restriction enzyme sites were underlined.

| **Primer** | **Sequence (5'-3')** |
| --- | --- |
| **RACE** | |
| *AjCTRP9* 3-1 | GAACCCATAGAGGACACCATACT |
| *AjCTRP9* 3-2 | AACAAATGGTCCAATCAGAACGA |
| **Real-time PCR** | |
| miR-31 | AGGCAAGATGTTGGCATAGCT |
|  | TaKaRa miScript universal primer |
| RNU6B | CGTGAAGCGTTCCATATTTTAA |
|  | TaKaRa miScript universal primer |
| *AjCTRP9* qF | TCCAGGCAGAACCCATAGAG |
| *AjCTRP9* qR | TATCCGGCAGTGGAAGACA |
| *Ajcaspase-3* qF | TCAGGGACTACTTTGATGGATGG |
| *Ajcaspase-3* qR | TGTGTTGGTGGGGTTGGAATG |
| *Ajcaspase-6* qF | AGAATGAACAGGAGAGTCGGAAC |
| *Ajcaspase-6* qR | TGAGTGAGAAAAGCACACAGGAA |
| *Ajcaspase-8* qF | GGAGATGGACAGGCGTTCTTTAC |
| *Ajcaspase-8* qR | CGATACCGTCCTTGTGGAACTCT |
| *Ajserine palmitoyltransferase 1* qF | CTGTTTCTCAGCCTCACTGCCTC |
| *Ajserine palmitoyltransferase 1* qR | CAACGGATAAAACCAACCCTCGG |
| *Ajserine palmitoyltransferase 2* qF | CCGAGACCAACTGCGAAGACT |
| *Ajserine palmitoyltransferase 2* qR | AACTCAGCACCTGGGACACCT |
| *Ajceramide synthase 1* qF | AGACATCCGAGAGGACGACGAG |
| *Ajceramide synthase 1* qR | GTCACTCCTTCTGGCTTTGCGT |
| *Ajceramide synthase 5* qF | ACTGTATGTTTGCTGGTTTCGC |
| *Ajceramide synthase 5* qR | TCGCTTCGTATGTCTTTCTCGT |
| *Ajceramide synthase 6* qF | TCCAGAAAATGTATCGTGGGGTG |
| *Ajceramide synthase 6* qR | TTCGTGGGAACTCCAAGAGCAAT |
| *Ajneutral ceramidase-like 1* qF | TTGGTCCTAACACACTACGAGCG |
| *Ajneutral ceramidase-like 1* qR | CTTCAGGGTCAGCAAGAGTGGTT |
| *Ajneutral ceramidase-like 2* qF | AGAATGTGGCTATTTGTGGGACG |
| *Ajneutral ceramidase-like 2* qR | GAGATTGTCATAGGCATCGTGGA |
| *Ajputative neutral sphingomyelinase* qF | GCAGGAACAGGACGGTGAACT |
| *Ajputative neutral sphingomyelinase* qR | TCAAAGAACGCTTCGTAACCG |
| *Ajβ-actin* F | CCATTCAACCCTAAAGCCAACA |
| *Ajβ-actin* R | ACACACCGTCTCCTGAGTCCAT |
| **Vector construction** | |
| *AjCTRP9* 3'UTR-MluI F | ACGCGTgaggaagactaaaccagtccag |
| *AjCTRP9* 3'UTR-HindIII R | AAGCTTGTGTCCCACAAAAATAGTTACTT |
| *AjCTRP9* 3'UTR- mutation F | GATCGTATGTCATTTACGGAATTGTAATT |
| *AjCTRP9* 3'UTR- mutation R | CCGTAAATGACATACGATCTGCTCTCGAT |
| *AjCTRP9-*EcoRI F | GAATTCCGTGTTCACGAGGAGACCCT |
| *AjCTRP9-*NotI R | GCGGCCGCATGGAGTTGTAGCCAGACC |
| **siRNA synthesis** | |
| *AjCTRP9* siRNA F1 | CCAGCUACAGUACUAGAAATT |
| *AjCTRP9* siRNA R1 | UUUCUAGUACUGUAGCUGGTT |
| *AjCTRP9* siRNA F2 | UUUCUAGUACUGUAGCUGGTT |
| *AjCTRP9* siRNA R2 | AUGCUCUUUAAACGGAUCCTT |
| *AjCTRP9* siRNA F3 | AUGCUCUUUAAACGGAUCCTT |
| *AjCTRP9* siRNA R3 | AUGCUCUUUAAACGGAUCCTT |
| Negative control (NC) | UUCUCCGAACGUGUCACGUTT  ACGUGACACGUUCGGAGAATT |
| **miRNA mimics and inhibitors** | |
| miR-31 mimics | AGGCAAGAUGUUGGCAUAGCU  CUAUGCCAACAUCUUGCCUUU |
| miR-31 mimics negative control (NCM) | UUCUCCGAACGUGUCACGUTT  ACGUGACACGUUCGGAGAATT |
| miR-31 inhibitor | AGCUAUGCCAACAUCUUGCCU |
| miR-31 inhibitor negative control (NCI) | CAGUACUUUUGUGUAGUACAA |
